# Lodgings for Women

**Published:** 1911-06-03

**Authors:** 


### Lodgings for Women.

It is to be hoped that the National Conference which the Lord Mayor opened on Wednesday, May 18, for the discussion of the provision of good and suitable lodgings for women of every class, will not, as so many previous conferences have done, pass away without results. The industrial conditions of our age make it imperative that many women should leave the shelter of their parents' roof, and it is of the highest importance to the State that such women should be able to find decent, comfortable, sanitary lodgings at a reasonable rate and within a reasonable distance of their work. The protest against the "living-in" system in the drapery trade, which has developed so much of late years, is an increasing inducement for those who are truly interested in the welfare of women to find for girls who would otherwise have been housed and fed—perhaps not under altogether ideal conditions, but still with a certain amount of security—another, and if possible a better, place of residence.

The Duchess of Marlborough, in her opening address, poured some contempt upon the argument that to provide decent homes for working women is to destroy home life. It is inevitable that many women—most of them young and at an age when careful supervision is important—must leave their homes in order to make their living. They do not in any sense of wilful flight leave their homes. They are, since they must earn their bread, forced to leave them, and the question is, what feasible imitation of a home, what sanitary environment, what comfort in health, what care in sickness, we are prepared to provide for them? It may frankly be admitted that at the best we cannot do all that is wanted—the best of States cannot be a mother—but have we done what we could?

The answer must be in the negative. Cheap lodgings, supervised, and thus kept up to a certain point of decency, by municipal authority, have been provided for men, but almost nothing for women. Yet for women a decent lodging is a more important asset than it is for men. An employer is less apt to ask a man if he is living at home or to be anxious about his environment than he is when he is engaging a woman. Respectability counts more with a woman than with a man. Her private life, even if not subject to supervision, must be passed in a place where it can be supervised. And yet, with a constantly increasing number of women who are seeking employment, we have done but little to make it possible for them to fulfil the conditions upon which we employ them. To live in a common lodging-house is looked upon as discreditable, while yet the earnings of many workers forbid their living in any better quarters. If there were in London, as there is in Glasgow, a lodging for women only, many of the unsavoury suggestions which now surround the idea would vanish. There is no reason why such a place should not be at least self-supporting, even if it did not show a profit, and the gain to women would be enormous.

While it is perhaps the poorest who suffer most from the lack of cheap, clean, and respectable accommodation, the demand for this runs through every class of women workers. Some blocks of flats are built and set apart for women in London, and these are in great demand, although they are for the most part expensive, and some are lacking in convenience. But they touch only the fringe of the demand and provide for only a limited class, and the same may be said of residential clubs. There are thousands of women who need accommodation of a cheaper kind, which shall yet be sanitary and respectable, and which will combine such reasonable supervision as will make it a place where a mother may be willing to send her daughter with an equally reasonable amount of freedom. The women of to-day are self-reliant, and neither need nor will endure the grandmotherly limitations which were common in the older experiments in homes for women; but there are some rules which every self-respecting woman will gladly observe. It should not pass the wit of man—or at any rate of woman—to plan and organise such homes and hostels as are needed by the various classes. It remains to be seen whether or not it will be beyond the enterprise of municipal bodies or private philanthropists to provide them.

